# Computational Approaches for the Analysis of ncRNA through Deep Sequencing Techniques

**DOI:** 10.3389/fbioe.2015.00077

**Published:** 2015-06-03

**Authors:** Dario Veneziano, Giovanni Nigita, Alfredo Ferro

**Affiliations:** ^1^Department of Molecular Virology, Immunology and Medical Genetics, The Ohio State University, Columbus, OH, USA; ^2^Department of Clinical and Molecular Biomedicine, University of Catania, Catania, Italy

**Keywords:** RNA-seq, miRNA, lncRNA, circRNA, bioinformatics

## Abstract

The majority of the human transcriptome is defined as non-coding RNA (ncRNA), since only a small fraction of human DNA encodes for proteins, as reported by the ENCODE project. Several distinct classes of ncRNAs, such as transfer RNA, microRNA, and long non-coding RNA, have been classified, each with its own three-dimensional folding and specific function. As ncRNAs are highly abundant in living organisms and have been discovered to play important roles in many biological processes, there has been an ever increasing need to investigate the entire ncRNAome in further unbiased detail. Recently, the advent of next-generation sequencing (NGS) technologies has substantially increased the throughput of transcriptome studies, allowing an unprecedented investigation of ncRNAs, as regulatory pathways and novel functions involving ncRNAs are now also emerging. The huge amount of transcript data produced by NGS has progressively required the development and implementation of suitable bioinformatics workflows, complemented by knowledge-based approaches, to identify, classify, and evaluate the expression of hundreds of ncRNAs in normal and pathological conditions, such as cancer. In this mini-review, we present and discuss current bioinformatics advances in the development of such computational approaches to analyze and classify the ncRNA component of human transcriptome sequence data obtained from NGS technologies.

## Introduction

For over five decades, the central dogma of molecular biology has represented the basis of genetics (Crick, 1970), essentially describing the genetic information flow of life in which DNA and protein, as respectively repository and functional incarnation of that information, have been viewed as the two main actors in the life of the cell, confining RNA simply to the role of template for protein synthesis. Nevertheless, this view of the biological role of RNA, initially apparently exhaustive, has been over time subjected to challenges, as firstly suggested by Gilbert in 1986 (Gilbert, 1986).

As interest on the hypothesized “RNA world” grew, subsequent studies allowed to explore the potential of such new vision (Lee et al., 1993; Fire et al., 1998), eventually leading to one of the most significant biological discoveries of the past decade: the existence of several types of RNAs, each with their specific functions in eukaryotic cells (Eddy, 2001; Todd and Karbstein, 2007). As the ENCODE project has confirmed, most of the human genome is in fact transcribed, but only a very small fraction of it encodes for proteins (Birney et al., 2007; Elgar and Vavouri, 2008). Indeed, the larger remaining portion of the transcribed genomic output is represented by a diverse family of untranslated transcripts that play crucial roles in many biochemical cellular processes (Mattick, 2001).

These non-coding RNAs (ncRNAs) are divided into two major categories most commonly according to their nucleotide sequence length: small (<200 bp) and long (200 bp or more). Within each category, there are several distinct classes, each one with its own three-dimensional folding and specific function.

From the more popular classes of small structural ncRNAs, such as transfer RNAs (tRNAs) and ribosomal RNAs (rRNAs), focus has shifted in the last 10 years to a set of small RNA classes involved in post-transcriptional regulation: microRNAs (miRNAs) whose precursors (pre-miRNAs) form a peculiar hairpin structure; small interfering RNAs (siRNAs); piwi-interacting RNAs (piRNAs).

Growing interest has more recently emerged also toward long ncRNAs (lncRNAs) which constitute the majority of the non-protein-coding transcripts (Ponting et al., 2009). Having length >200 nt, lncRNAs, already thought of potentially regulating transcription via chromatin modulation, may be also involved in post-transcriptional regulation, organization of protein complexes, and cell–cell signaling (Meldrum et al., 2011).

Finally, an additional class of ncRNAs is represented by circular RNAs (circRNAs) which have been proven to be untranslated, very stable, abundant, and conserved RNA molecules in animals (Jeck et al., 2013).

Yet, despite having been more than a decade since the human genome was sequenced, most transcribed regions are still of unknown molecular function and biological significance.

A potential approach to solving this problem is provided by the ever increasing application of high throughput sequencing technology (HTS), also known as next-generation sequencing (NGS). In fact, numerous transcriptomic sequencing projects are accumulating with increasing rapidity, generating data which are enabling the identification of different types of ncRNAs, and the quantification of their expression levels in different tissues, conditions, and developmental stages.

## Why NGS?

Deep sequencing provides a very promising tool. NGS can produce millions of sequences at lower cost in shorter time than before (Meldrum et al., 2011) delivering greater sensitivity and accuracy than previous technologies. Its sensitivity and specificity are above microarray techniques (‘t Hoen et al., 2008; Wang et al., 2009); it does not rely on target probe hybridization, permitting the sequencing of the exact transcript on a single nucleotide resolution (Zhou et al., 2011), thus allowing the identification of variations in length or composition (Jung et al., 2010); it requires no previous transcript information (Isakov et al., 2012), utilizing any relevant database to compare and characterize the sequence population (Ronen et al., 2010); it provides high depth of coverage for any library of nucleic acids and it can be modified to study specific properties, e.g., small RNA-seq (sRNA-seq) (Landgraf et al., 2007); it can be used on species for which a full-genome sequence is not yet available; RNA editing events can be detected, and knowledge of polymorphisms can provide direct measurement of allele-specific expression (Malone and Oliver, 2011).

Several HTS platforms are commercially available, each characterized by specific data throughput, read length, error rate, and price (Zhou et al., 2011), providing a wide choice of options.

## Current Computational Approaches for ncRNA Analysis from NGS Output

Earlier attempts at whole genome identification of ncRNAs generally had already focused on distinct expression patterns and novel RNA structural families to better characterize the properties of ncRNAs. An example of this is the *incRNA* pipeline employed by Lu et al. (2011) who have developed a comprehensive machine-learned model integrating sequence, structure, and large-scale expression data, both deep sequencing and array. This proves how the complementary nature of combined features can clearly separate ncRNAs from other genomic elements and potentially differentiate between distinct ncRNA types, representing an important advantage of integrative approaches.

Such characterization studies have provided methods that can be adapted to different organisms to identify novel ncRNAs from unannotated genomic regions, paving the way for the development of integrated tools.

Moreover, the large amount of data generated by HTS experiments has made it absolutely necessary to dispose of bioinformatics methods in order to properly store, analyze, and visualize such data.

Generally, a ncRNA bioinformatics analysis system can be comprised of three essential components: a post-sequencing data analysis pipeline for ncRNA detection, classification and expression analysis representing the core of the system; a data module to provide annotation information and storage for the analysis results; a visualization/query system for viewing and functionally analyzing raw data and elaborated results.

As proven by Cordero et al. (2012), statistical detection of differential expression of NGS data gives efficient results when computational strategies employ statistical models based on NB distribution [i.e., baySeq (Hardcastle and Kelly, 2010)] or on variance [i.e., DESeq (Anders and Huber, 2010), DESeq2 (Love et al., 2014)], as opposed to non-parametric methods which are frequently used for microarray-generated data but are very sensitive to background composition when applied to NGS data.

In order to satisfy the urgent demand for intuitive and efficient data exploration and relieve the growing pressure on handling massive quantities of short-read sequences, several NGS-based RNA transcriptome bioinformatics analysis tools/pipelines have been developed (Tables 1 and 2), and below we give an overview of the current most popular ones.

**Table 1:** **Small non-coding RNA Tool comparison**.

		miRDeep	miRDeep*	miRSpring	DARIO	CPSS	ncPRO-seq	CoRAL	RNA-CODE
Package	Online server				**√**	**√**	**√**		
	Stand-alone	**√**	**√**	**√**			**√**	**√**	**√**
Applicable to	Raw data	**√**	**√**			**√**	**√**		**√**
	Mapped data		**√**	**√**	**√**		**√**	**√**	
Input format	FASTQ/FASTA	**√**	**√**			**√**	**√**		**√**
	BAM/SAM		**√**	**√**	**√**		**√**	**√**	
	BED				**√**				
	GFF/GTF							**√**	
Assembly	*De novo*								**√**
	Reference genome sequence	**√**	**√**	**√**	**√**	**√**	**√**	**√**	
Known miRNA detection		**√**	**√**	**√**	**√**	**√**	**√**	**√**	**√**
Known other ncRNA detection					**√**	**√**	**√**	**√**	**√**
Novel ncRNA prediction		**√**	**√**		**√**		**√**	**√**	
Expression analysis		**√**	**√**	**√**	**√**	**√**	**√**	**√**	**√**
miRNA target prediction			**√**			**√**			
miRNA target functional enrichment						**√**			

**Table 2:** **Long non-coding RNA Tool comparison**.

		CoRAL	RNA-CODE	lncRScan	iSeeRNA	CIRI	Annocript	LncRNA2Function
Package	Online server				**√**			**√**
	Stand-alone	**√**	**√**	**√**	**√**	**√**	**√**	
Applicable to	Raw data		**√**				**√**	**√**
	Mapped data	**√**		**√**	**√**	**√**		
Input format	FASTQ/FASTA		**√**			**√**	**√**	**√**
	BAM/SAM	**√**				**√**		
	BED				**√**			
	GFF/GTF	**√**		**√**	**√**	**√**		
Assembly	*De novo*		**√**				**√**	
	Reference genome sequence	**√**		**√**	**√**	**√**		**√**
Known miRNA detection		**√**	**√**					
Known other ncRNA detection		**√**	**√**					
Novel ncRNA prediction		**√**		**√**	**√**	**√**	**√**	**√**
Expression analysis		**√**	**√**	**√**	**√**	**√**		

### Small ncRNA transcription investigation approaches

Throughout the last decade, the study of the small RNA transcriptome has been gradually recognized to be essential to fully comprehend the complex scenario of transcriptional regulation. For this reason, most currently available tools/pipelines for transcriptome investigation through NGS concentrate on detection/prediction/expression quantification of small RNAs, especially miRNAs.

*miRDeep* (Friedländer et al., 2008) is believed to be the first stand-alone tool used to analyze large-scale sRNA-seq data in order to detect both known and novel miRNAs. miRDeep employs Bayesian probability controls along the steps of miRNA biogenesis to estimate the false-positive rate and the sensitivity of predictions. The algorithm assumes that if a read is truly related to a pre-miRNA, then it must be a portion either of the loop sequence or of one of the potential two mature sequences in the hairpin. Thus, given the higher abundance of the dominant mature sequence in the cell compared to any other sequence of a pre-miRNA, the higher number of reads in the data will likely correspond to mature sequences, while less frequent reads may map to other parts of the hairpins. Algorithms for mapping and evaluation of free energy, previously under user control, are carried out by Bowtie and Randfold in miRDeep2 (Bonnet et al., 2004; Langmead et al., 2009; Friedländer et al., 2012) in which species conservation has been a key addition as well (Mackowiak, 2011).

Modeled off miRDeep, mirDeep* (An et al., 2013) employs a miRNA precursor prediction strategy which the authors have proved to outperform both versions of miRDeep as it adopts a different strategy to excise the potential precursor locus range, resulting in a lower number of false negatives. Users can also apply the original miRDeep prediction algorithm, as well as the TargetScan (Lewis et al., 2005) algorithm in order to predict targets for identified known and novel miRNAs.

Great innovation in terms of portability and the elaboration of miRNA processing information is provided by the *miRspring* software (Humphreys and Suter, 2013). The tool generates a small portable interactive miRNA Sequence Profiling document capable of completely reproducing all the information from a significantly larger mapped sequencing data file in bam format (i.e., from a miRNA-Seq experiment), along with providing miRNA processing statistics. In fact, it is the first software that allows to visualize the processing features, seed distribution and relative expression levels of genomic clustered miRNAs from a whole miRNA data set.

Aside miRNA-specific approaches, other software focuses on small RNAs in general.

The first integrated tool ever developed for the analysis and prediction of several classes of small ncRNAs on RNA-seq data originating from arbitrary sequencing platforms is the web service *DARIO* (Fasold et al., 2011). The software provides a straighforward interface which allows users to quantify ncRNAs in a completely platform independent way. DARIO annotates reads with information provided by several ncRNA public databases, and excludes mapping loci overlapping with exonic regions, while setting apart those that overlap with introns and intergenic regions for non-annotated ncRNA prediction. An extension of this system to plants has recently been published (Patra et al., 2014).

The web server *CPSS* (Zhang et al., 2012) takes things a step further. The tool can analyze small RNA deep sequencing data coming from single or two paired samples, with special emphasis on miRNAs. Data are classified into several categories of small ncRNAs according to several referred annotations. Matched mapped reads are then quantified for expression analysis (differential in case of two samples), while unmatched ones are employed to predict novel miRNAs also through miRDeep (Friedländer et al., 2008). CPSS also provides users with the possibility to predict target genes for differentially expressed novel/known miRNAs but, like no precedent approach, it also performs functional enrichment analysis of those targets for further experimental or computational studies.

Differently, *ncPRO-seq* (Chen et al., 2012), a stand-alone, comprehensive and flexible ncRNA analysis pipeline, systematically investigates all small ncRNA species in a given annotation family in an unbiased way, providing the user with detailed descriptions of read distribution. Furthermore, the tool defines novel small ncRNA families by identifying regions significantly enriched with short reads not classified under any known ncRNA species, allowing the discovery of previously unknown ncRNA- or siRNA-coding regions.

To address the limitations of RNA function prediction methods in classifying ncRNA classes, the machine learning package *CoRAL* (Leung et al., 2013) classifies RNA transcripts from sRNA-seq data into functional categories by relying on biologically interpretable features more informative than sequence or alignment information, like certain aspects of small RNA biogenesis. Leveraging on the assumption that such biological properties should be consistent within classes of ncRNAs sharing the same molecular function (i.e., across different tissues or organisms), CoRAL was trained in order to identify the most informative features in regard to the molecular mechanisms and metabolic processes of each functional ncRNA class. Based on fragment length, cleavage specificity, and antisense transcription, CoRAL can effectively classify six distinct ncRNA classes among miRNAs and transposon-derived RNAs. Outperforming previous tools such as DARIO and miRDeep2, CoRAL provides the opportunity to annotate ncRNAs in other less well-characterized organisms.

Another tool for ncRNA annotation in NGS data lacking reference genomes is the software *RNA-CODE* (Yuan and Sun, 2013). As ncRNA homology search takes advantage of both sequence and secondary structure similarity, optimization for NGS data is still widely absent, especially when a reference genome is missing. To compensate for this, RNA-CODE combines secondary structure based homology search with *de novo* assembly, adjusting the assembly parameters in a family specific fashion. The sofware assumes that true ncRNA reads sequenced from the same gene can be assembled into contigs with significantly high alignment scores against their native families, while reads aligned by chance tend to share poor overlaps and thus are not likely to be assembled. Sensitivity and accuracy of short reads classification is thus greatly improved. Biogenesis-based properties and homology search results are instead employed for ncRNAs, such as miRNAs, which could not as easily be assembled into contigs. The classification results can then be used to quantify the expression levels of different types of ncRNAs, both small and long, in RNA-seq data of non-model organisms.

### Circular RNA detection algorithms

The works done by the Brown (Salzman et al., 2012) and Sharpless (Jeck et al., 2013) groups are forerunners of a series of algorithmic approaches to effectively identify circRNA, attempting to compensate the non-uniformity of RNA-seq data sets.

Most algorithms have focused on junction read detection whether leveraging on annotated exon boundaries (Salzman et al., 2012), adopting a two-segment alignment for split reads (Memczak et al., 2013) or relying on RNAase-treated sequencing (Jeck et al., 2013). Nevertheless, all these methods are annotation-dependent and unable to detect certain types of circRNAs having complex alignments and/or subject to experimental bias.

A very recent computational tool proven to outperform any precedent approach in the detection of circRNAs from NGS is *CIRI* (Gao et al., 2015). CIRI is an unbiased, annotation-independent approach employing a *de novo* algorithm able to accurately detect novel circRNAs based on paired chiastic clipping (PCC) signals combined with a filtering system able to remove false positives. CIRI has been able to specifically identify for the first time the prevalence of intronic/intergenic circRNAs as well as fragments specific to them in the human transcriptome, providing novel targets for further functional studies.

### Long ncRNA transcription investigation approaches

Long ncRNA investigation is a challenging task, as many more NGS reads are required to achieve adequate coverage compared to mRNAs or other types of ncRNAs. Here below, we describe a few recent computational tools which very well represent the general approach employed by several studies so far (Guttman et al., 2010; Cabili et al., 2011; Pauli et al., 2012).

The pipeline employed by Sun et al. (Sun et al., 2012) makes use of a software they have specifically developed to detect novel lncRNA, called *lncRScan*. The pipeline aims at tackling three of the major technical problems encountered in studying lncRNAs through RNA-seq: eliminating partial transcripts and artifacts in the assembled transcriptome due to RNA-seq-specific issues; identifying lncRNA from the complexity of assemblies; distinguishing lncRNAs from protein-coding mRNAs. After mapping and assembly, the data obtained are compared to a set of combined gene annotations in order to maximize detection and facilitate category labeling of novel transcripts, retaining only multi-exon ones not possessing any annotation for downstream processing. After quality control, the remaining assemblies are given as input to lncRScan for novel lncRNA detection. The tool identifies the candidate lncRNAs through a five-step filtering process: first it organizes input transcripts into five broad categories according to their genomic location in relation to annotated gene transcripts; transcripts longer than 200 nt are selected, filtering out those with open reading frame (ORF) >300 nt; in the last two steps, phylogenetic analysis and potential aminoacidic sequences of the remaining transcripts are performed in order to exclude any protein-coding potential. Performance evaluation of the pipeline has shown its great ability to filter out mRNAs from the candidate set, while revealing a stringent prediction of true lncRNAs from a test set.

*iSeeRNA* (Sun et al., 2013) is an SVM-based classifier which can accurately and quickly identify lincRNAs from large datasets, employing conservation, ORF- and nucleotide sequences-based features in order to appropriately distinguish lincRNAs from protein-coding transcripts (PCTs). The best classification results on test sets were produced leveraging on 10 features from the three categories mentioned above: sequence conservation score, being lincRNA less conserved than PCTs in general; ORF length and ORF proportion compared to transcript total length; frequencies of seven di- or tri-nucleotide sequences. Homolog search-based features were instead not included due to lack of annotation for novel PCTs which could foster misclassification. Trained in a species-dependent manner, iSeeRNA allows the user, however, to build additional customized SVMs for other species of interest. Supporting file formats widely used by the RNA-Seq assemblers, iSeeRNA can be easily integrated into transcriptome data analysis pipelines.

More recently, Soreq et al. (Soreq et al., 2014) have integrated full profile characterization of lncRNAs into their comprehensive RNA-seq analysis workflow. The pipeline, based on sample specific database construction, is able to analyze count information from RNA-seq data originating from several platforms and mapping analysis methods. After sequence reads have been mapped, their genome coordinates are intersected with those of the largest available database of reconstructed transcript models for lncRNAs, GENECODE (Derrien et al., 2012). Following appropriate filtering, differential expression of the detected lncRNA candidates is performed using the Bioconductor edge-R package (Robinson et al., 2010) which accounts for biological and technical variability as well as moderating the degree of over-dispersion across transcripts, thus improving the reliability of the results.

Musacchia et al. (2015) provide instead a pipeline combining the identification of both coding and long non-coding RNAs in *de novo* generated transcriptomes, without the support of comparative data. *Annocript* identifies putative lncRNAs by leveraging on public annotation databases and sequence analysis software to verify lack of protein/domain similarity, lack of long ORFs, and high non-coding potential.

Finally, an innovative approach in the functional annotation of lncRNAs is provided by Jiang et al. (2015). *LncRNA2Function* provides the first ontology-driven user-friendly web system based on the idea that similar expression patterns across multiple conditions may share similar functions and biological pathways. The tool functionally annotates a single or a set of lncRNAs with the functional terms significantly associated to the set of protein-coding genes significantly co-expressed with the lncRNAs. Standard mapping and assembly are thus followed by the computation of Pearson Correlation Coefficients for all lncRNA–mRNA gene pairs, assigning to each lncRNA a set of significantly co-expressed protein-coding genes which provides the lncRNA with functional and pathway annotations significantly enriched in such set. The tool thus allows to browse the results obtained from an RNA-seq dataset of 19 human normal tissues in order to retrieve the set of lncRNAs associated to a specific functional term, the set of functional terms associated to a lncRNA or assign functional terms to a set of lncRNAs, thus providing a precious resource for lncRNA function investigation.

## Conflict of Interest Statement

The authors declare that the research was conducted in the absence of any commercial or financial relationships that could be construed as a potential conflict of interest.
